# Mesenchymal stem cells and their therapeutic applications in inflammatory bowel disease

**DOI:** 10.18632/oncotarget.16682

**Published:** 2017-03-29

**Authors:** Fei Mao, Qiang Tu, Li Wang, Fuliang Chu, Xia Li, Haiyan S. Li, Wenrong Xu

**Affiliations:** ^1^ Key Laboratory of Medical Science and Laboratory Medicine of Jiangsu Province, School of Medicine, Jiangsu University, Zhenjiang, Jiangsu, P.R. China; ^2^ Jiangning Hospital of Nanjing, Nanjing, Jiangsu, P.R. China; ^3^ Department of Lymphoma and Myeloma, The University of Texas MD Anderson Cancer Center, Houston, Texas, USA; ^4^ Department of Gastroenterology, Binzhou Medical University Yantai Affiliated Hospital, Yantai, Shandong, P.R. China; ^5^ Department of Immunology, The University of Texas MD Anderson Cancer Center, Houston, Texas, USA

**Keywords:** mesenchymal stem cell, inflammatory bowel disease, cell therapy, pericyte, tissue repair

## Abstract

Mesenchymal stem or stromal cells (MSCs) are non-hematopoietic stem cells that facilitate tissue regeneration through mechanisms involving self-renewal and differentiation, supporting angiogenesis and tissue cell survival, and limiting inflammation. MSCs were originally identified and expanded in long-term cultures of cells from bone marrow and other organs; and their native identity was recently confined into pericytes and adventitial cells in vascularized tissue. The multipotency, as well as the trophic and immunosuppressive effects, of MSCs have prompted the rapid development of clinical applications for many diseases involving tissue inflammation and immune disorders, including inflammatory bowel disease. Although standard criteria have been established to define MSCs, their therapeutic efficacy has varied significantly among studies due to their natural heterogenicity. Thus, understanding the biological and immunological features of MSCs is critical to standardize and optimize MSCs-based therapy. In this review, we highlight the cellular and molecular mechanisms involved in MSCs-mediated tissue repair and immunosuppression. We also provide an update on the current development of MSCs-based clinical trials, with a detailed discussion of MSC-based cell therapy in inflammatory bowel disease.

## INTRODUCTION

Mesenchymal stem or stromal cells (MSCs) represent a heterogeneous population of stromal cells that have self-renewal ability and multipotent differentiation potential. The presence of non-hematopoietic stem cells in the bone marrow was first proposed by Cohnheim about 150 years ago; who demonstrated the origination of wound-repairing cells from the distal bloodstream [1]. In 1968, Tavassoli and Crosby found that transplantation of intact bone marrow pieces into extramedullary sites in rodents could reconstitute both hematopoietic and adventitial structures [2], providing further evidence for the existence of non-hematopoietic stem cells.

The definitive discovery of MSCs has generally been credited to Friedenstein, who reported a plastic-adherent cell subpopulation from the bone marrow or spleen that retained colony-forming activities and could differentiate into osteoblasts *in vitro* and *in vivo* upon transplantation in the 1970s [3–5]. The term MSCs was not introduced until 1991 by Arnold Caplan, who defined MSCs as stromal cells that are “capable of differentiating through a series of separate and unique lineage transitions into a variety of end-stage phenotypes” [6]. MSCs possess the abilities of self-renewal, tissue migration, and multipotency; they constitute tissue cells in the bone, cartilage, and fat. In addition, they can influence tissue repair *via* paracrine effects or direct cell-to-cell contact. Thus, the use of MSCs as potential cell therapy for a variety of diseases has been extensively explored, and the number of clinical trials of MSCs has risen nearly exponentially in recent years.

Inflammatory bowel disease (IBD), including ulcerative colitis (UC) and Crohn's disease (CD), is a chronic disease of the gastrointestinal tract that is characterized by perpetual idiopathic intestinal inflammation. IBD is more prevalent in western countries with an estimated rate of 0.5%, and its prevalence is rapidly increasing in Asian countries. The etiology of IBD is unclear but involves a multifactorial interactions among genetic susceptibility, dysregulated immune responses, and environmental factors. Chronic inflammation in IBD is well known to predispose patients to colitis-associated cancer. Anti-inflammatory approaches, such as tumor necrosis factor (TNF) inhibitors, blocking antibodies against the interleukin (IL)-6 pathway, and Janus kinase inhibitors, have been actively evaluated to determine their efficacy in IBD treatment. With the rapid advances in MSC research, efforts have been made to investigate the therapeutic potential of MSCs in IBD. In this review, we discuss the mechanisms by which MSCs contribute to tissue repair and their applications in IBD treatment in experimental animals and patients.

## IDENTIFICATION, ORIGIN, AND DIVERSITY OF MSCS

For many years since their discovery, MSCs have only been identified in cultures of developed organs based on their plastic adherence, phenotypic and functional characteristics. To standardize MSCs from different sources, the International Society of Cell Therapy specified three minimal criteria for MSCs in 2006: plastic adherence in culture, specific phenotypic markers (CD105^+^ CD73^+^ CD90^+^ CD45^-^ CD34^-^ CD14^-^ CD19^-^ HLA-DR^-^), and the ability to differentiate into osteoblasts, adipocytes, and chondroblasts *in vitro* (Table 1). However, the extensive use of culture-based MSCs has raised some doubts about their native identity and anatomic distributions due to concerns on their phenotypic changes during *in vitro* expansion [7–11].

**Table 1:** Key characteristics of MSCs

FEATURES	DESCIPTION
Native precursor	Pericytes and adventitial cells
Morphological characteristics	CD105^+^ CD73^+^ CD90^+^ CD45^-^ CD34^-^ CD14^-^ CD19^-^ HLA-DR^-^
Phenotype	Plastic adherent
Plasticity	Multipotent
Differentiation potential	Osteoclasts, adipocytes, chondroblasts, etc.
Tissue origin	Vascularized tissues (bone marrow, adipose tissue, umbilical cord), pluripotent stem cells
Preparation methods	Long-term culture, sort-purification

Multiple research groups have identified or isolated MSC-like cells from the vascular walls of distinct human organs [12–15], leading to the proposition that MSCs reside at perivascular sites. In 2008, Crisan *et al*. demonstrated that cultured CD146^+^ vascular pericytes (CD146^+^ CD34^-^ CD45^-^ CD56^-^) from multiple human tissues exhibited features closely resembling those of culture-derived MSCs, providing the first evidence of tissue-resident MSCs. Other studies have reported that pericytes or perivascular precursors directly contribute to the development and regeneration of odontoblasts in dental pulp [16], skeletal muscle [17], and follicular dendritic cells [18], confirming their functional similarity to MSCs *in situ*. CD34^+^ adventitia cells (the outermost layer of blood vessels) were also found to express MSC markers and retain pluripotency [19–21]. Adventitia cells do not express typical pericyte markers such as CD146, αSMA, PDGFRβ, and NG2; however, these markers could be upregulated in response to angiopoietin-2. These observations led to the hypothesis that MSCs are indeed adventitial cells and precursors of pericytes [19]. The identification of pericytes and adventitial cells as true MSCs has been confirmed by a sequential series of transplantation experiments, in which a single MSC generates a transplantable clonal progeny in recipients [21, 22] (Table 1). These results are consistent with previous findings that cells with MSC characteristics have been identified in virtually all vascularized tissue and organs, including bone marrow, adipose tissue, and liver, and can be culturally expanded *ex vivo* [23].

In addition, increasing evidence shows that pluripotent stem cells (PSs), including embryonic stem cells (ESCs) and induced pluripotent stem cells, can efficiently develop into cells with MSCs features *via* epithelial-to-mesenchymal transition (extensively reviewed in [24]) (Table 1). MSCs derived from vascularized tissue and PSs show no major differences in regard to their surface markers, differentiation potential, or immunotolerogenic ability [25–31]. However, PS-derived MSCs inherit some features of their pluripotent progenitors, as they have faster proliferation rates than do tissue-derived MSCs, which make them more attractive for experimental and clinical use. Kimbrel *et al*. demonstrated that intraperitoneal delivery of PSs-derived MSCs significantly improved the survival of lupus-prone BWF1 mice and reduced the severity of experimentally induced uveitis [29]. Contemporaneously, another study using a mouse experimental autoimmune encephalomyelitis model showed that human ESCs-derived MSCs had better therapeutic performance in reducing symptoms and neuronal demyelination than bone marrow-derived MSCs [32].

With recent insights into the perivascular origin of MSCs and advances in flow cytometry techniques, MSCs can be freshly isolated from vascularized tissues to avoid traditional cell culture-based MSCs enrichment (Table 1). Adipose tissue is one of the most dispensable and easily accessible sources of perivascular MSCs; approximately 3-15 × 10^6^ MSCs can be isolated per 100 ml of lipoaspirate, which provides sufficient amounts for clinical applications [33, 34]. In addition, native MSCs can be directly purified from bone marrow, umbilical cord, and other tissues but are often limited by low yield and unstable donor supply sites for clinical applications [35, 36]. MSCs from heterogeneous resources fulfill the common criteria of MSCs but differ in terms of immunophenotypes and biological potency, thus introducing variations and discrepancies in therapeutic efficacy among studies [35, 36]. While traditional methods of MSC isolation rely on long-term culture, which is not only time-consuming but may also alter the phenotypic and functional properties of MSCs [7–11]; the purification of native MSCs is an alternative and neater cell source in regenerative therapy.

Of note, MSCs often represent a mixed population of cells with distinct phenotypic and biological properties. Increasing numbers of studies have used surface markers to further classify MSC subsets with respect to proliferation and survival rates, immunomodulatory features, and differentiation preference. A recent review by Mo *et al*. clearly addressed this issue [35]. External factors, such as isolation technique, culture conditions, cell source, and donor origin, can also influence the bioactivity and fate of MSCs *in vivo* [37]. Thus, understanding MSCs’ heterogeneity and optimizing their isolation and expansion will significantly aid in the selection of MSCs for therapeutic advantages for different conditions.

## MECHANISMS INVOLVED IN MSCs-MEDIATED TISSUE REPAIR AND IMMUNOSUPPRESSION

**Figure 1:**
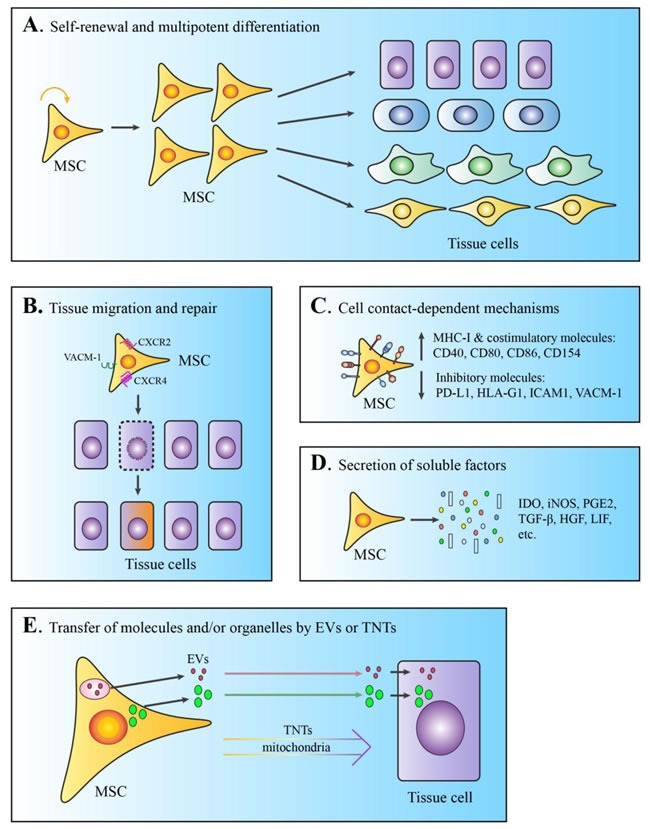
Schematic demonstration of the diverse mechanisms involved in MSCs-mediated tissue repair **A**. Self-renewal and multipotent differentiation. **B**. Tissue migration and repair or replacement of damaged tissue cells. **C**. Cell contact-dependent immunosuppressive functions *via* surface molecules. **D**. Immunotolerance and angiogenesis mediated by secretion of soluble factors. **E**. Transfer of molecules or organelles by EVs or tunneling nanotubes (TNTs). All the demonstrated MSCs-mediated functions can be modulated by bioactive or inflammatory reagents, such as nitric oxide, IFN-γ, and TNF-α.

Tissue homing and tissue regeneration. Early studies by Friedenstein and many others clearly established that plastic-adherent MSCs are multipotent and readily develop into a variety of specialized tissue lineages *ex vivo*, such as osteoblasts, chondrocytes, adipocytes, and myoblasts [3–6, 38–42] (Figure 1A). With the optimization of techniques involved in the expansion and isolation of large quantities of MSCs in culture, studies have further characterized the behavior of these cells *in vivo*. MSCs are able to migrate and seed specifically into damaged tissue sites, where they can differentiate into functional cells to replace damaged or diseased cells [43–53] (Figure 1B). Trafficking-related molecules, such as CCR2, CXCR4, VCAM1, and MMP2, have been suggested to facilitate MSC homing [54–59]. Upon their arrival at inflamed tissue, some MSCs developed into myofibroblasts in an experimental rat colitis model, with upregulated expression of α-SMA and desmin [60]. Although MSCs are capable of replacing damaged tissue cells *via* self differentiation *in situ*, their tissue repair properties are largely due to their ability to stimulate the proliferation and survival of local tissue while inhibiting tissue cell apoptosis and fibrosis through the secretion of proteolytic enzymes and angiogenic factors [61–64]. Bioactive reagents, such as nitric oxide, IFN-γ, or TNF-α, can also motivate MSCs’ functions by altering their migration, differentiation, or immunologic properties, as detailed below [55, 65–67]. The degree and duration of MSCs’ engraftment is a crucial factor affecting the efficacy of tissue repair and regeneration.

### Cell-intrinsic or cell contact-dependent immunotolerogenic properties

MSCs lack the major MHC class II molecule and express only low levels of MHC class I and costimulatory molecules (CD40, CD80, CD86, and CD154) (Figure 1C). This unique feature of surface markers is generally considered as a hypoimmunogenic phenotype, which allows MSCs to evade immune recognition and clearance upon *in vivo* delivery. A recent review by Ankrum *et al*. argues against MSCs’ immunoprivileged status, as several experimental and clinical studies have reported that allogeneic MSCs can elicit both innate and adaptive immune responses in recipient hosts [68]. Nevertheless, rejection of allogeneic MSCs occurs much more slowly than does rejection of other allogeneic cell types, such as fibroblasts [69]. The coinhibitory molecule PD-L1 has been found to be constitutively expressed, or to be induced by IFN-γ, on human and mouse MSCs derived from bone marrow, liver, and placenta [70–72] (Figure 1C). Using monoclonal antibodies or siRNAs, studies have revealed that increased amounts of surface PD-L1 are associated with MSC-mediated inhibitory effects on T cell proliferation and cytotoxicity *in vitro*.

HLA-G1 is another cell surface molecule with a similar inhibitory function, as the addition of HLA-G1-blocking antibodies to MSC:T cell coculture nearly restored T cell proliferative responses to anti-CD3/CD28 stimulation [73] (Figure 1C). Similar approaches have demonstrated that the adhesion molecules ICAM-1 and VACM-1 are also involved in MSC-mediated immunotolerance, although to a lesser extent [74] (Figure 1C). In addition, bone marrow-derived MSCs express high levels of surface FasL, which directly induces T cell apoptosis and facilitates the production of T cell-chemoattractive MCP-1 [75]. Of note, accumulating evidence indicates that the “reprogramming” of MSCs by proinflammatory cytokines derived from immune cells is necessary for their efficacy [67]. Thus, the balance between proinflammatory *versus* suppressive factors in the *ex vivo* culture and *in vivo* microenvironment greatly influences the function and duration of MSCs engraftment [68].

### Secretion of immunomodulatory mediators

Besides their cell-intrinsic functions, MSCs are known to produce a wide spectrum of trophic factors that are critical in the suppression of immune responses and tissue regeneration (Figure 1D). By using neutralizing antibodies in a series of mixed lymphocyte reaction assays, Di Nicola *et al*. proposed that TGFβ1 and hepatocyte growth factor were the major soluble mediators responsible for the MSCs-mediated reduction in T cell proliferation [76]. Other research groups have suggested that the suppressive effects of MSCs largely depend on elevated prostaglandin E2 (PGE2) levels, as inhibitors of PGE2 efficiently abolished MSCs-mediated immunosuppression [77, 78]. Aggarwal and Pittenger demonstrated that MSC-derived PGE2 elicited a shift in T cell polarization from Th1 to Th2 subtype, while inducing dendritic cells to produce more IL-10 at the expense of reduced secretion of TNF-α and IL-12 [77]. Constitutive expression of TGFβ, hepatocyte growth factor, and PGE2 in human MSCs was confirmed separately by Ryan *et al* [79].

The expression of the tryptophan-degrading enzyme, indoleamine-2, 3-dioxygenase (IDO), was also involved in the suppressive effects of MSCs in an IFN-γ-dependent manner [79, 80]. Similarly, IFN-γ elicited the production of large quantities of inducible nitric oxidase synthase in MSCs, which is required for their immunosuppression [81]. Both inducible nitric oxidase synthase and IDO gene expression is dependent on IFN-γ-mediated STAT1 activation, suggesting that STAT1 is critical for the immunosuppressive function of MSCs [51]. In addition, MSCs were reported to repress the activity of macrophages, dendritic cells, and T cells *via* TNFα-stimulated gene protein 6 by activating the NF-κB signaling axis downstream of CD44 receptor [82–84]. Stimulation of MSCs with TLR agonists poly I:C and LPS, IFN-γ, or TNF-α was reported to induce the expression of the above-mentioned factors [79, 82, 85–87]. Other molecules, such as leukemia inhibitory factor, galectin, and insulin-like growth factor, are also believed to participate in the immunoregulatory network of MSCs [88–90].

### Transfer of bioactive materials by extracellular vesicles

The term “extracellular vesicles (EVs)” generally refers to small phospholipid vesicles released by most, if not all, somatic cell types; they can be further classified as exosomes (30-100 nm), microvesicles (50-1000 nm), and apoptotic bodies (1-5 μm). Once released to the extracellular milieu, EVs can be picked up by other cells, leading to the intercellular exchange of bioactive components such as proteins, lipids, and nucleic acids. Exosomes are formed inside multi-vesicular bodies and released following fusion with the plasma membrane; they comprise a homogenous population. Microvesicles, on the other hand, are released by direct shedding from the plasma membrane and comprise more heterogeneous vesicles. Exosomes and microvesicles are better characterized EVs in MSCs and are referred to together as EVs in this review hereafter.

In 1997, Timmers *et al*. discovered that systemic delivery of MSCs-conditioned medium containing large compounds of 50-100 nm markedly reduced infarct size and improved cardiac performance in a porcine model of ischemia and reperfusion injury [91]. Three years later, the same group identified EVs as the active component in supernatants of human ESC-derived MSCs, which provided cardioprotection [92, 93]. MSC-derived EVs (MSC-EVs) have been investigated in different experimental settings and rodent models (Figure 1E). *in vitro* studies demonstrated that they exert their function directly on a wide range of immune cells involved in innate and adaptive immune responses, including monocytes, dendritic cells, B cells, and different T cell subsets (reviewed in [94]). In parallel, the results of numerous studies using animal models of organ injury indicate that MSC-EVs have great therapeutic benefits for injured hearts, livers, lungs, kidneys, brains, skin, and other organs and tissues by increasing tissue cell proliferation, reducing cell death, and limiting inflammation (reviewed in [93, 95]). MSC-EVs are highly enriched in small RNA molecules, such as miRNA, which have been demonstrated to mediate their beneficial effects [96–100]. In addition, they deliver protein- and peptide-based paracrine effectors, such as cytokines, hormones, and transcription factors, which also participate in MSC-mediated tissue repair [101–104]. In Burrello *et al*'s review, mRNAs, transcription factors, and cytokines are the major effectors identified up date that play important roles in MSC-EV-mediated immunosuppression [104]. Future studies are required to fully elucidate the molecular content and mechanisms used by EVs to mediate their function.

#### Transfer of mitochondria by tunneling nanotubes

Mitochondria generate the majority of metabolic energy (ATP) in eukaryotic cells through respiration and oxidative phosphorylation. Intercellular transfer of mitochondria has been suggested to occur through tunneling nanotubes, which can rescue cells with impaired mitochondria function [105–107]. MSCs are proficient mitochondrial donors and express high levels of RHOT1, a key RhO GTPase that supports mitochondrial transport from MSCs to adaptor cells [108]. Mitochondrial transfer has been found between MSCs of different sources and a variety of damaged tissue cells *ex vivo* [109] (Figure 1E). Furthermore, recent studies have provided compelling evidence that mitochondrial transfer from administered MSCs to injured airway epithelial cells or vascular endothelial cells facilitates tissue regeneration and reduces organ function [107, 108, 110, 111]. One study further demonstrated that the capability of mitochondrial transfer among different MSCs was associated with their therapeutic effects in a cigarette smoke-induced model of lung injury [107]. Of note, mitochondrial dynamics and trafficking in MSCs are probably regulated by many signaling cascades beyond RHOT1 [106, 109]; thus, strategies that improve the mitochondrial transfer of MSCs may further improve MSC-based cell therapy.

## THERAPEUTIC APPLICATIONS OF MSCS IN IBD AND OTHER DISEASES

Inflamed tissue damage and dysregulated immune response are the major pathogenic features of IBD, and MSCs use the molecular and cellular mechanisms discussed above in IBD treatment. An increase in effector T helper (Th) subset activation (Th1/Th17 in CD and Th2 in UC) has been documented, along with reduced regulatory T cell (Treg) levels in circulation in IBD patients [112–115]. These activated T cells are more resistant to apoptosis because of an imbalance of the anti- and pro-apoptotic Bcl-2 family proteins Bcl-2, Bcl-x(L), and Bax [116–118]. Human or autologous MSCs, regardless of their origin and route of administration, have been demonstrated to engraft into inflamed intestinal and mesenteric lymph nodes in rodent models of colitis induced by dextran sodium sulfate or trinitrobenzene sulfonate, with documented tissue persistence time varying from 3 to 15 days [62, 119–121]. One study showed that a small proportion of transplanted MSCs was able to develop into colonic interstitial myofibroblasts [60]. Another study demonstrated that the systemic infusion of bone marrow MSCs promoted the differentiation and proliferation of intestinal epithelial cells, as evidenced by a remarkable increase in Ki67 and Lgr5 in damaged colonic tissue [62]. In these studies and some others, treatment with MSCs was able to suppress Th1/Th17 cells and boost Treg cells in mesenteric lymph nodes, accompanied by a systemic reduction in pro-inflammatory cytokines (IFN-γ, IL-17, IL-6, and TNF-α) and an elevation of anti-inflammatory cytokine IL-10 [75, 119–122].

In addition, intravenous administration of bone marrow MSCs was reported to induce T cell apoptosis *via* FasL-Fas engagement in murine models of dextran sodium sulfate-induced colitis and fibrillin-1-mutated systemic sclerosis [75]. Phagocytosis of dead T cells by macrophages in turn triggered TGF-β production, which led to enhanced Treg differentiation [75]. In the murine colitis model and other experimental settings, MSCs were shown to directly inhibit the antigen-presenting function of dendritic cells and macrophages, rendering them toward tolerogenic phenotype with increased IL-10 secretion and favored Treg induction [123–125]. More recently, in a trinitrobenzene sulfonate rat colitis model, the intravenous injection of MSC-EVs substantially reduced colonic damage and NF-κB activities, accompanied by decreased proinflammatory cytokines and increased IL-10 [126]. Collectively, these findings indicate that MSCs exert stemness and immunosuppressive functions in animal models of colitis, which cooperatively suppress pathogenic T cells and reduce the disease phenotype.

**Figure 2:**
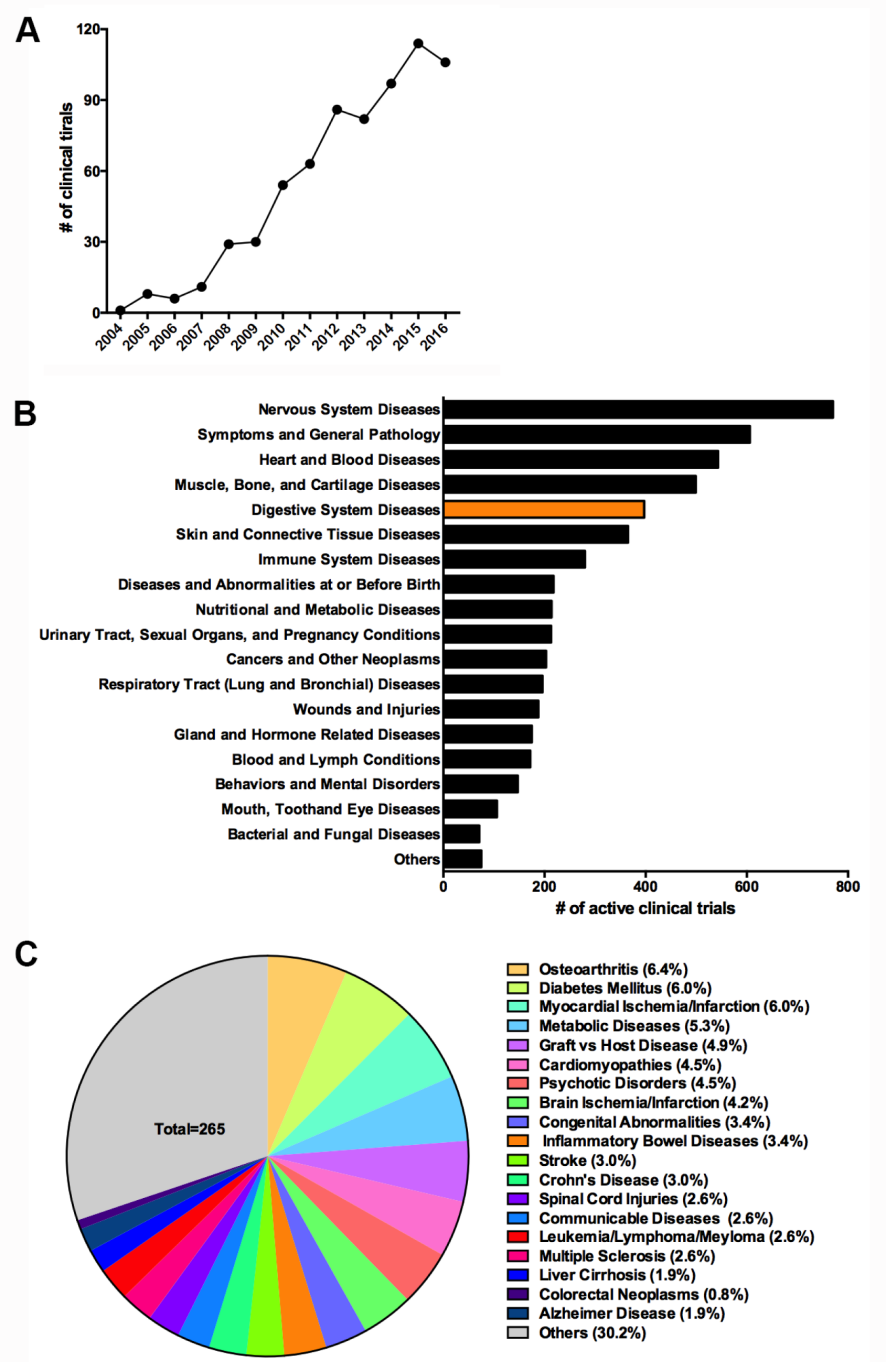
MSCs-based clinical trials on clinicaltrials.gov **A**. Number of registered clinical trials of MSCs-based therapy from 2004 to 2016 (as of December 25, 2016). **B**. Distribution of ongoing MSCs-based therapy by disease category. **C**. Distribution of ongoing MSCs-based therapy by selected common diseases.

The self-renewing, multipotency, and immunosuppressive potentials of MSCs have prompted increased clinical investigations of their use in the treatment of connective tissue diseases and immune disorders, including IBD, graft-*versus*-host disease, and autoimmune disease. We searched the clinicaltrials.gov database using the terms “mesenchymal stem cell” and “mesenchymal stromal cell” and retrieved a total of 687 clinical trials, with a nearly exponential increase since 2004 (Figure 2A). On the basis of disease categories used by clinicaltrials.gov, diseases of the nervous system, heart and blood; muscle, bone, and cartilage; skin and connective tissue; and immune system comprise more than 50% of ongoing clinical trials (total = 265), with digestive tissue disease ranked fifth (Figure 2B). More specifically, IBD and colorectal neoplasms together are being studied in approximately 4.2% of ongoing clinical trials (Figure 2C). Although the niche function of bone marrow MSCs in supporting hematopoiesis has been recognized, the clinical applications of MSCs have been limited and only a few clinical trials have been conducted in hematopoietic cancers (Figure 2C).

In a phase I clinical study involving four patients with fistulising CD, one intrafistular inoculation of autologous MSCs from adipose tissue culture (3-30 × 10^6^) resulted in healing in 6 of 8 fistulas and reduced drainage in 2 of 8 fistulas at 8 weeks. The authors observed no adverse effects after 12-30 weeks’ follow-up [127]. In the expanded phase II trial of 49 patients with complex cryptoglandular fistulas or CD, the combination of fibrin glue and two doses of adipose-derived MSCs (20 × 10^6^) resulted in significantly enhanced efficacy in comparison to the control group (17 of 24 *vs* 4 of 25 for fistula healing, RR = 4.43, *p* < 0.001), with no MSC-related adverse effects [119]. Comparable results were obtained in studies from a separate group using similar MSCs generation protocols in patients with perianal fistulas associated with CD [128, 129]. Autologous MSCs derived from the bone marrow were only evaluated in 10 patients in a phase I clinical trial. The authors demonstrated that repeated intrafistular inoculation of MSCs greatly improved clinical remission (7 of 10 for complete closure and 3 of 10 for incomplete closure of fistula openings), accompanied by increased local and systemic Tregs [130].

Local administration of allogeneic MSCs in patients with refractory CD and complex fistulas achieved similar therapeutic benefits as autologous MSCs, although some adverse effects (i.e., anal abscess and uterine leiomyoma) were reported [131–133]. In these studies, MSCs were expanded from liposuctioned adipose tissue or bone marrow aspirates from healthy donors. Separately, Chinnadurai *et al*. reported that MSCs derived from CD patients were functionally similar to those of healthy controls in terms of T cell inhibition, and their immunosuppressive properties were dependent on IFN-γ responsiveness and IDO [80]. In contrast, Zhu *et al*. demonstrated that although gastric cancer tissue-derived MSC-like cells share many characteristics with bone marrow MSCs, they exhibit a stronger cancer-promoting capability, a phenotype that is dependent on miR155-5p-mediated NF-kB signaling [134]. More detailed comparisons between MSCs from different sources, especially those from inflamed or diseased tissues, are necessary for a better understanding of the microenvironment's influence on MSCs’ characteristics.

The therapeutic effects of autologous MSCs on luminal IBDs *via* systemic administration are contradictory. One study showed improved clinical outcomes with adipose-derived MSCs [135]; two found that a large majority of patients experienced no clinical improvement or even worsening of the disease with bone marrow-derived MSCs [136, 137]. The inefficiency of MSC-based therapy on luminal IBDs could be because high percentages of MSCs were localized in the lungs after intravenous injection [138, 139]. Surprisingly, better results were obtained from trials using allogeneic MSCs expanded in cell culture of bone marrow or umbilical cord, where significant reduction in disease severity and clinical remission occurred in more than half the patients [140, 141]. While all the completed clinical trials with published results were performed with conventional tissue culture-derived MSCs, recent advances in sort-purification from adipose tissues allow the enrichment of large quantities of native MSCs for clinical evaluation. Collectively, it is critical to assess the safety and efficacy of MSC therapy related to origin, dose, route of administration, and other modification strategies to further improve MSC-based clinical applications.

## CONCLUDING REMARKS

The roles of MSCs as direct mesenchymal progenitors, anti-inflammatory modulators, and tissue niche cells not only present opportunities but also challenges to fully understanding the complexity of their physiological roles *in situ*. While the rapid translation of the results of biological studies of MSCs to therapy is important, more stringent criteria or a more strict definition of MSCs seems necessary to enforce standard clinical applications. As the function of MSCs is tightly influenced by the microenvironmental milieu, it is not surprising that MSC-based therapy does not always confer therapeutic benefits. Indeed, MSCs have been shown to exert pro-tumorigenic effects in cancers, including colorectal cancer, by promoting cancer cell proliferation, formation of the tumor-associated stromal network, and angiogenesis [142, 143]. Thus, serious efforts are needed to further investigate and characterize the precise mechanisms involved in MSC-based tissue remodeling in a disease- and tissue-specific manner. In addition, optimized standards for MSCs isolation and expansion, delivery dose, and safety control are also required to establish intra-study or intra-country comparisons.
